# c-Myc-Regulated lncRNA-IGFBP4 Suppresses Autophagy in Cervical Cancer-Originated HeLa Cells

**DOI:** 10.1155/2022/7240646

**Published:** 2022-08-29

**Authors:** Lei Zhang, Zongshan Zhang, Erwei Li, Poshi Xu

**Affiliations:** ^1^Department of Laboratory Medicine, Henan Provincial People's Hospital, Zhengzhou, Henan, China; ^2^Department of Laboratory Medicine of Central China Fuwai Hospital, Central China Fuwai Hospital of Zhengzhou University, Zhengzhou, Henan, China; ^3^Department of Blood Transfusion, Henan Provincial People's Hospital, Zhengzhou, Henan, China; ^4^Department of Blood Transfusion of Central China Fuwai Hospital, Central China Fuwai Hospital of Zhengzhou University, Zhengzhou, Henan, China

## Abstract

LncRNAs are known to regulate a plethora of key events of cellular processes; however, little is known about the function of lncRNAs in autophagy. Here in the current study, we report lncRNA-IGFBP4 which has previously been known to regulate the proliferation and reprogramming of cancer cells, but its role in autophagy is not yet known. We found that serum starvation provokes autophagy-induced downregulation of lncRNA-IGFBP4 levels. Next, we determined that c-Myc can negatively regulate lncRNA-IGFBP4 in HeLa cells. Phenotypically, we found that upon depletion of lncRNA-IGFBP4, the HeLa cells undergo autophagy through ULK1/Beclin1 signaling. Furthermore, through TCGA data analysis, we found lncRNA-IGFB4 overexpressed in most cancers including cervical cancer. Based on these findings, we conclude that c-Myc maintains cellular homeostasis through negatively regulating lncRNA-IGFBP4 in cervical cancer cells.

## 1. Introduction

Autophagy is a biological phenomenon that keeps balance in the cellular homeostasis through the lysosomal degradation [1], and it plays a very crucial role in many biological processes and pathologies including cancers, inflammation, immune response, neurological disorders, and metabolic diseases [2, 3]. Recently, different molecular factors have been known to regulate autophagy in cancer cells [4, 5], including different proteins, epigenetic factors, and RNAs especially lncRNAs [6]. For long noncoding RNAs (lncRNAs), transcripts more than 200 bp without no protein-coding ability have attracted great attention in the past decade [7] due to their regulatory roles in various molecular and biological pathways [6, 8] and gene regulation either transcriptionally or posttranscriptionally [9]. LncRNAs are considered as one of the key players in cancer regulation, genetic alterations, or expressional variations in lncRNAs which significantly impact the cancer cell growth, cellular apoptosis, metastasis, immune regulation, or vice versa [10–13]. A bulk of lncRNAs have been identified and are associated with cancer development and being validated as molecular markers for cancer prognosis, metabolism, and metastasis such as lncRNA LINC-PINT as a key factor in the regulation of various cancers [7], lncRNA MALAT-1 as metastasis marker [14], and lncRNA TRINGS nutrition stress-associated lncRNAs [15].

As mentioned earlier, autophagy is a very crucial process for cellular homeostasis involving various factors including lncRNAs, but not many lncRNAs have been identified in autophagy. However, some of the lncRNAs are known to potentially regulate autophagy such as lncRNA-FA2H-2 [16], lncRNA SOX2 [17], and lncRNA CAIF [18], and mostly these lncRNAs induce factors associated with inflammation and immune system or other common oncopathways [19]. Similarly, in the current study, we identified a lncRNA insulin-like growth factor binding protein 4–1 (IGFBP4–1) which has been known to potentially regulate cellular proliferation and cancer cell [20]. It serves as an oncogene and a tumor suppressor in some cancers, and upon overexpression, the proliferation rate of cancer cells abruptly increases in the lung cancer [21] and shows opposite effects in the prostate, colon, and breast cancers [22–24]. This lncRNA is related to the gene IGFBP4 which is a significant member of IGFBP family proteins; it inhibits tumor growth by inhibiting IGF-induced metabolic processes [22]. It has been found upregulated in a variety of cancers such as lung, pancreatic, gastric, and kidney cancers [25–27] suggesting an oncogenic role as well as high metastasis and poor prognosis [28]. Given that, no study has been conducted on the role of IGFBP4-1 in autophagy and cervical cancer development. Therefore, in the current study, we specifically determined the role of IGFBP4-1 in autophagy, and subsequently, we analyzed its prognostic and molecular function in cervical cancer cells.

## 2. Materials and Methods

### 2.1. Reagents and Antibodies

The reagents and antibodies used in this study are purchased from the indicated manufacturers: Myc-inhibitor (sigma), c-Myc (Santa Cruz), GAPDH (Santa Cruz), LC3 (Santa Cruz), Actin (sigma), P62 (Santa Cruz), ULK1 (Santa Cruz), Beclin1 (Santa Cruz), Flag (Sigma), and HRP-conjugated secondary antibodies (Promega). The primers and oligos are enlisted in supplementary Table S1.

### 2.2. Cell Culture and Transfections

HeLa and 293 T cell lines were cultured in Dulbecco's modified Eagle's medium (DMEM) with a glucose concentration of 25 mM and supplemented with 10% fetal bovine serum (FBS) (Gibco), 1% penicillin/streptomycin, and 1% sodium pyruvate at 37°C with 5% CO_2_. For serum starvation, we used DMEM including other supplements except for FBS. For transient transfections, we used lipo8000 (Beyotime) according to the manufacturer's instructions.

### 2.3. RNA Interference and lncRNA-IGFBP4 Overexpression

We cotransfected 293 T cells cultured in a 6-cm dish with pLKO.1-shRNA (2 *μ*g), pREV (2 *μ*g), pGag/Pol/PRE (2 *μ*g), and pVSVG (1 *μ*g) to generate lentiviral particles. The next day, the old medium was replaced with a fresh DMEM medium, and cells were allowed to culture for 48-72 h. Subsequently, we collected the medium through filtering them with 0.45-um filters. HeLa cells were incubated with a medium containing the lentiviral particles supplemented with 10 *μ*g/ml polybrene (Sigma). After 12-16 h later, the infection was removed, cells were washed with PBS, and a fresh medium was added. 48 h after infection (transduction), the cells were selected with 5*μ*g puromycin-supplemented DMEM and thereafter cultured in DMEM containing puromycin. This was followed by the determination of the knockdown efficiency of lncRNAs-IGFBP4 by qPCR. Similarly, we overexpressed lncRNA-IGFBP4 (cloned in pSin vector) and transfected 293 T cells for lentivirus production, followed by infection in HeLa cells, puromycin selection, transfection efficacy, and further analyses.

### 2.4. RNA Isolation and Real-Time Quantitative PCR

The RNA from respected cell lines was isolated using an RNA isolation kit (Zymo Research, R2050) and following the manufacturers' instructions, then the quantity and quality of RNA were determined by nanodrop. A total of 1ug RNA was used to synthesize using random hexamers and oligo (dT) primers by the iScript™ cDNA synthesis kit (Bio-rad#1708891). RNA expression was measured by quantitative real-time polymerase chain reaction (qRT-PCR) using the ABI Prism 7500 System (Applied Biosystems) with cycling conditions of 95°C for 10 min, followed by 40 cycles of 95°C for 15 s and 60°C for the 60s. All the qPCR experiments were conducted in triplicates, and the results were analyzed through the 2^-*ΔΔ*Ct^ method; the relative expression of RNA was normalized by taking *β*-actin as an internal control. The list of primers (forward and reverse) used in this study was given in Supplementary Table S1.

### 2.5. Western Blot Analysis and Coimmunoprecipitation

For western blot analysis, the cells were lysed using RIPA buffer (Abcam#ab156034), and proteins were quantified using the BCA method. Next, we added 2× Protein Laemmli buffer (Biorad#1610737EDU) and boiled the samples for 10 mins. A total of 20*μ*g of protein was loaded onto 10-12% SDS-PAGE followed by standard western blot analysis. The high-quality images of immunoblots were captured with Thermo Scientific™ machine.

### 2.6. Luciferase Assay

To determine the effect of transcription factor c-Myc on the lncRNA-IGFBP4 promoter, HEK293T cells were transfected with control plasmid or Flag-c-Myc together with the pGL3-based construct containing lncRNA-IGFBP4 wild-type promoter or promoter with mutated sites plus Renilla luciferase plasmid. In short, the HEK293T cell were cultured in DMEM medium supplemented with 10% FBS; after achieving 70-80% cell growth, the cells were cotransfected with the dual-luciferase reporter, corresponding lncRNA, target, and negative controls using Lipofectamine 2000. Next, after 24 h, the luciferase reporter activity was measured by using a luciferase assay kit (Promega) and plotted after normalizing concerning Renilla luciferase activity (mean ± SD).

### 2.7. Chromatin Immunoprecipitation (ChIP) Assay

The HeLa cells were cultured in a 10-cm dish and transfected with a 3x-Flag-c-Myc or empty vector. After 24 h when cells reached 90% confluency, the cells were cross-linked with 1% formaldehyde for 10 mins. Next, the cells were washed with PBS and collected in Eppendorf, followed by the addition of RIPA buffer. DNA fragments with approximately 300–1,000 base pairs (bp) in length were produced by shearing the DNA with help of sonication. Subsequently, we centrifuged the lysates for 10 min using a refrigerated ultracentrifuge at 12,000 g at 4°C, and supernatants were collected in a new tube. ChIP assay was performed by the Pierce Agarose ChIP kit (Thermo Scientific, USA) using Flag antibody (Sigma) according to manufacturer's instructions. Finally, the bound DNA fragments were analyzed by real-time qPCR. The specific primers are included in Supplementary Table S1.

### 2.8. Growth Curve and Colony Formation Assay

After the knockdown of the lncRNA-IGFBP4, a total of 2 × 10^4^ cells were plated into each well of the 12 well plates, and the rate of cell multiplication was measured by simply counting cells after every 24 hours and presented as a growth curve. Additionally, to analyze the effect of lncRNA-IGFBP4 knockdown on HeLa cells, we plated ~1 × 10^3^ cells into each well of a 6-well plate and cultured them for 14 days with regulator changing media every 24 h to maintain the required pH for cell growth. The colonies were fixed using 4% paraformaldehyde, stained with crystal violet, and counted to analyze the difference in cell growth with control cells.

### 2.9. The Universal Expression of lncRNA-IGFBP4

LncRNA-IGFBP4 was analyzed in a wide variety of tumors including CESC and correlation analysis with c-Myc was performed from TCGA datasets using the online GEPIA2 database tool [29].

### 2.10. Reproducibility of the Data

All the experiments and data included in this manuscript were validated by repeating thrice, and the microscopy images and western blots shown in the figures are representatives of three independent experiments.

### 2.11. Statistical Analysis

We performed Statistical analysis using Microsoft Excel software and GraphPad Prism to calculate the significant differences between results, and the *P* value ≤0.05 was considered statistically significant.

## 3. Results

### 3.1. Serum Starvation Inhibits lncRNA-IGFBP4 Expression

**Figure 1:**
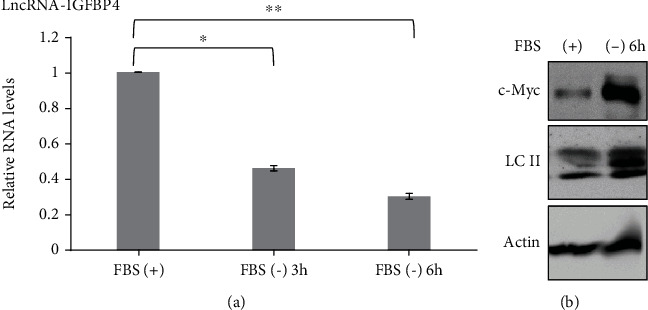
Serum starvation negatively regulates lncRNA-IGFBP4. (a) Expression levels of lncRNA-IGFBP4 in HeLa cells under serum-free growth conditions measured by qRT-PCR analysis. ±SEM, two-tailed Student *t*-test *n* = 3, ^∗^*P* < 0.05, ^∗∗^*P* < 0.001, ^∗∗∗^*P* < 0.0001. (b) Western blot analysis of c-Myc and autophagy marker LC3 under serum-free growth conditions. The images are representative of three independent experiments.

A previous study [21] indicated that lncRNA-IGFBP4 is involved in the reprogramming of energy metabolism. They showed that lncRNA-IGFBP4 could influence ATP production through changes in key enzymes (HK2, PDK1, and LDHA) in response to glucose analogue 2-DG [21]. This prompted us to evaluate the role of lncRNA-IGFBP4 in alternative energy-deficient conditions such as serum starvation. To this end, we used serum depleted growth medium to determine the levels of lncRNA-IGFBP4 at different time points. The HeLa cells were subjected to serum starvation conditions for indicated time points, and quantitative real-time PCR (qRT-PCR) analysis was performed. The results showed that lncRNA-IGFBP4 was downregulated when the growth medium was depleted from serum (Figure 1(a)). The downregulation of lncRNA-IGFBP4 in response to serum-free medium indicates that normal expression of lncRNA-IGFBP4 is important for cell proliferation and growth as highlighted in a previous study [21].

Therefore, based on the previous evidence and suggestion, we explored the role of lncRNA-IGFBP4 in serum-induced autophagy. We determined that autophagy was induced upon serum starvation, and the key regulator of energy metabolism in cancer, c-Myc, was also induced when cells were cultured in a serum-free growth medium (Figure 1(b)). These results allowed us to investigate if there is a connection between lncRNA-IGFBP4 and c-Myc.

### 3.2. c-Myc Negatively Regulates lncRNA-IGFBP4

**Figure 2:**
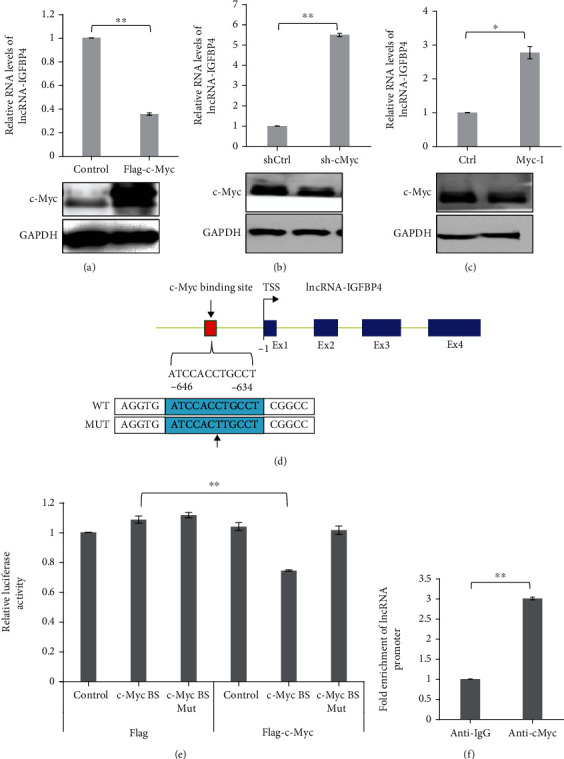
c-Myc negatively regulates lncRNA-IGFBP4. (a) lncRNA-IGFBP4 expression in control and HeLa cells with overexpression of flag-c-Myc analyzed by qRT-PCR analysis. ±SEM, two-tailed Student *t*-test *n* = 3, ^∗^*P* < 0.05, ^∗∗^*P* < 0.001, ^∗∗∗^*P* < 0.0001. Total protein extracts were subjected to western blot analysis against indicated antibodies. (b). lncRNA-IGFBP4 expression in control HeLa cells and cells depleted of c-Myc by shRNA-based lentiviruses, analyzed by qRT-PCR analysis. ±SEM, two-tailed Student *t*-test *n* = 3, ^∗^*P* < 0.05, ^∗∗^*P* < 0.001, ^∗∗∗^*P* < 0.0001. Total protein extracts were subjected to western blot analysis against indicated antibodies. (c) lncRNA-IGFBP4 expression in control and Myc-I treated HeLa cells, analyzed by qRT-PCR analysis. ±SEM, two-tailed Student *t*-test *n* = 3, ^∗^*P* < 0.05, ^∗∗^*P* < 0.001, ^∗∗∗^*P* < 0.0001. Total protein extracts were subjected to western blot analysis against indicated antibodies. (d) Graphical representation of c-Myc binding sites on the promoter of lncRNA-IGFBP4 with binding sequence and mutated sites. (e) Luciferase reporter assay for relative luciferase activity of constructs with WT or mutant c-Myc binding sites. ±SEM, two-tailed Student *t*-test *n* = 3, ^∗^*P* < 0.05, ^∗∗^*P* < 0.001, ^∗∗∗^*P* < 0.0001. (f) ChIP analysis shows the binding enrichment of c-Myc at the promoter of lncRNA-IGFBP4. ±SEM, two-tailed Student *t*-test *n* = 3, ^∗^*P* < 0.05, ^∗∗^*P* < 0.001, ^∗∗∗^*P* < 0.0001.

To determine the fact that whether c-Myc affects the RNA expression levels of lncRNA-IGFB4, we overexpressed the flag tagged c-Myc in the HeLa cells and subsequently performed real-time quantitative PCR (qPCR) to measure levels of lncRNA-IGFB4. Interestingly, we found with the exogenous upregulation of c-Myc that the expression levels of lncRNA-IGFB4 were reduced by more than 65% (Figure 2(a)). To further complement these results, we silenced the expression of c-Myc in Hela cells by using shRNA-mediated knockdown. As expected, the loss of c-Myc in HeLa cells upregulated the expression levels of lncRNA-IGFB4 (Figure 2(b)). In addition to knockdown, we also used a c-Myc inhibitor (Myc-I) to recapitulate the effects of c-Myc depletion on lncRNA-IGFB4 expression and found similar results as we noted in shRNA-mediated depletion of c-Myc. The Myc inhibitor increased the levels of lncRNA-IGFB4 by ~3 folds (Figure 2(c)). These results confirm that c-Myc has a negative influence on the expression of lncRNA-IGFB4.

We further determined the possibility of a direct regulatory relationship between c-Myc and lncRNA-IGFB4. Using the publicly available database JASPAR, we screened the promoter of lncRNA-IGFB4 for possible c-Myc binding sites (BS). The promoter analysis of lncRNA-IGFB4 revealed the existence of a c-Myc binding site ~650 bp upstream of the transcription start site (Figure 2(d)). Additionally, the luciferase reporter assay with wild-type c-Myc BS and/or mutant c-Myc BS discovered that only wild-type BS constructs were responsive to ectopically expressed Flag-c-Myc overexpression, but not the mutant BS (Figure 2(e)). These results were complemented with ChIP-seq assay, where Flag-c-Myc was able to pull down the pieces of the lncRNA-IGFB4 gene when identified with qPCR analysis (Figure 2(f)). These findings support the notion that c-Myc physically interacts with the promoter of lncRNA-IGFB4 and inhibits its transcription.

### 3.3. lncRNA-IGFBP4 Suppresses Autophagy

**Figure 3:**
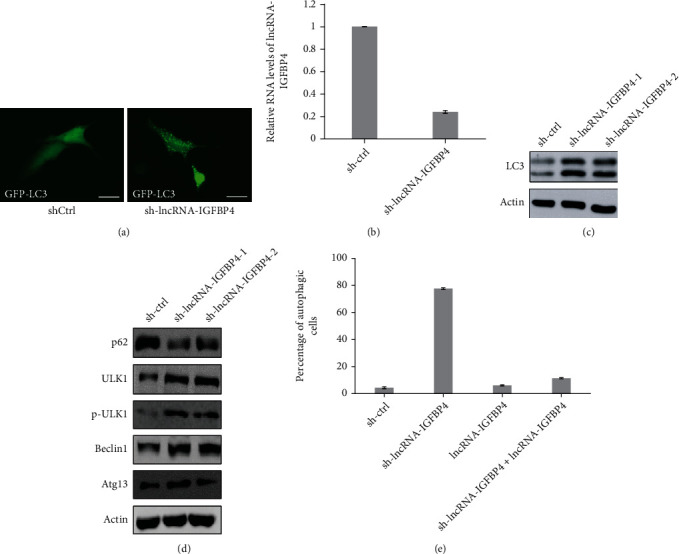
lncRNA-IGFBP4 suppresses autophagy in HeLa cells. (a) Fluorescent images of GFP-LC3 HeLa cells treated with control shRNA or shRNA targeted against lncRNA-IGFBP4. Images are representative of three independent experiments (scale bar: 10 *μ*m). (b) lncRNA-IGFBP4 expression in control and HeLa cells treated with shRNA targeted against lncRNA-IGFBP4, analyzed by qRT-PCR analysis. (c) Western blot analysis of autophagy marker LC3 of total cell lysates extracted from sh-control and shRNA targeted against lncRNA-IGFBP4. Actin was used as an internal control. Blots are representative of three independent experiments. (d) Western blot analysis of autophagy marker p62, ULK1, and Beclin1 of total cell lysates extracted from sh-control and shRNA targeted against lncRNA-IGFBP4. Actin was used as an internal control. Blots are representative of three independent experiments. (e) A high percentage of the autophagic cells was observed in the cells with depleted expression of lncRNA-IGFBP4 which were rescued by overexpression of lncRNA-IGFBP4.

Next, we sought to determine the effects of loss of functions for lncRNA-IGFB4 on cellular autophagy. For this purpose, we utilized the cells with shRNA-mediated depletion of lncRNA IGFB4. Surprisingly, we found that upon depletion of lncRNA-IGFB4, the cells undergo autophagy, as indicated by LC3 puncta formation in GFP-LC3 engineered HeLa cell line (Figure 3(a)). The knockdown efficiency of lncRNA is shown in Figure 3(b). We further confirmed the induction of LC3 by western blot, and the upregulation of LC3 protein in lncRNA-IGFB4-depleted cells confirmed the induction of autophagy (Figure 3(c)). In addition, we evaluated the changes in other known protein markers of autophagy such as the reduction in p62, and upregulation of Beclin1 and ULK1 (Figure 3(d)). Moreover, it has been reported that ULK1 phosphorylates Beclin1 and induces autophagy [30]. Here, we observed the upregulation of phosphorylated ULK1 (p-ULK1) in cells with depleted lncRNA-IGFB4 (Figure 3(d)), which shows that ULK1 phosphorylates the Beclin1 upon silencing of lncRNA-IGFB4..

Alternatively, in the rescue experiment, we overexpressed the lncRNA-IGFB4 in autophagy-induced HeLa cells, and we found that the exogenous overexpression of lncRNA-IGFB4 rescued the autophagic-positive cells which were initially induced by lncRNA-IGFB4-depletion (Figure 3(e)). These results support that lncRNA-IGFB4 inhibits autophagy in HeLa cells.

### 3.4. The Correlation of lncRNA-IGFBP4 and c-Myc in TCGA Cervical Cancer

**Figure 4:**
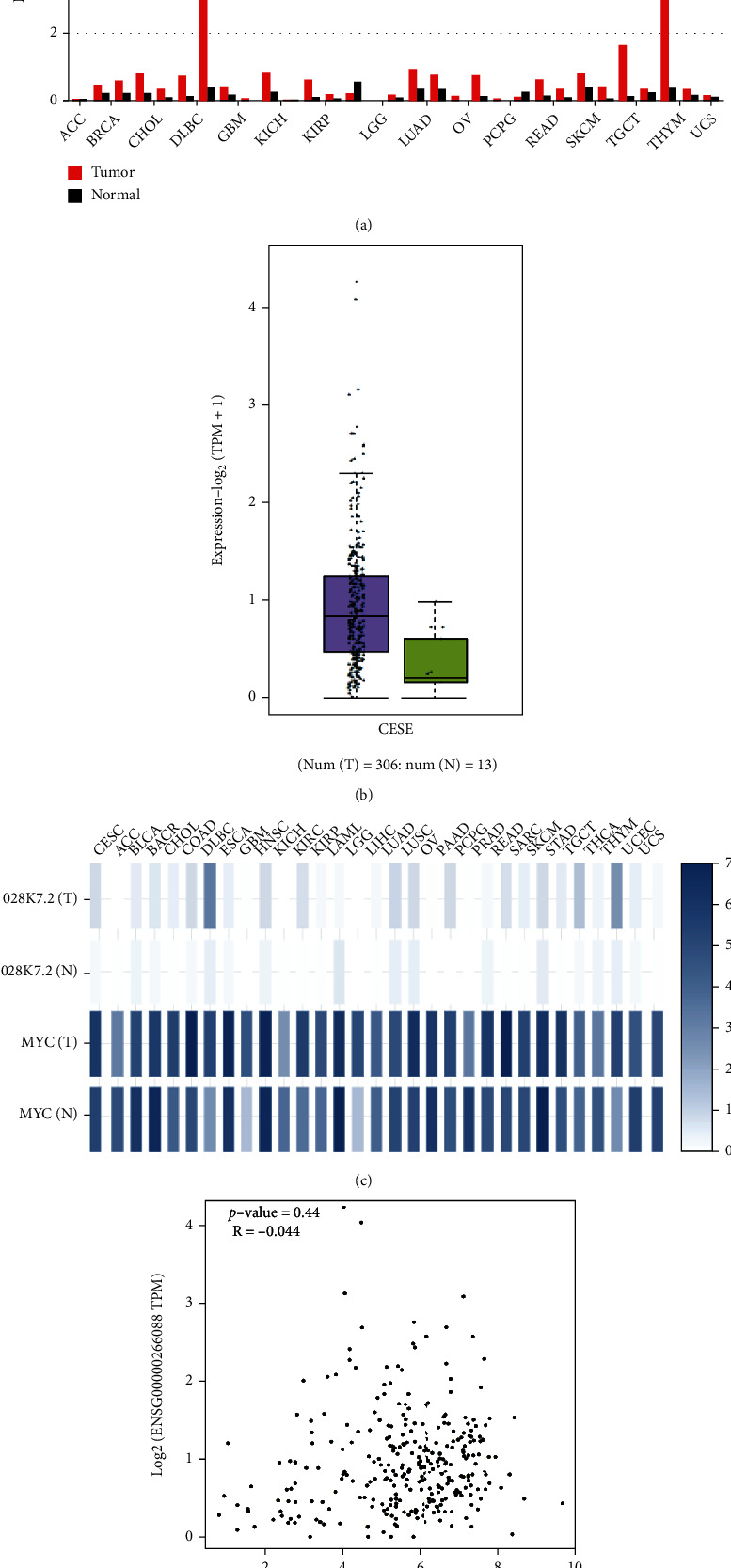
TCGA analysis of lncRNA-IGFBP4 expression. (a) lncRNA-IGFBP4 expression in TCGA paired samples. (b) Comparison of lncRNA-IGFBP4 expression in cervical cancer patients in TCGA profiles. (c) Heatmap of correlation of lncRNA-IGFBP4 and c-Myc expression in normal and tumor samples across TCGA sets. (d) Correlation analysis of lncRNA-IGFBP4 and c-Myc expression in cervical cancer (note: all the data in this figure were analyzed by using the online web tool GEPIA2 (https://gepia2.cancer-pku.cn/#index)).

Cervical cancer is the leading cause of death [18] and accounts for new cases [31] at higher rates among other gynecological tumors worldwide [32]. Therefore, we were interested to explore the involvement of lncRNA-IGFB4 in cervical cancer development. Our above results highlight lncRNA-IGFB4-mediated suppression of autophagy in a cervical cancer cell line, HeLa. Next, we investigated the expression levels of lncRNA-IGFB4 among all TCGA cancers particularly cervical squamous cell carcinoma and endocervical adenocarcinoma (CESC). For this purpose, we employed the publicly available database GEPIA2 and analyzed tumor samples from 32 TCGA cancers and GTEx normal samples. As shown in Figure 4(a), the expression of lncRNA-IGFB4 is upregulated in most TCGA cancers compared to the normal tissues. Next, we evaluated the expression of lncRNA-IGFB4 in 306 CESC tumors and 13 normal tissue samples. Our analysis uncovered that like other tumors, lncRNA-IGFB4 was upregulated in CESC too (Figure 4(b)). These results establish that lncRNA-IGFB4 is upregulated in most TCGA cancers including CESC.

As mentioned above, c-Myc negatively regulates the expression of lncRNA-IGFB4; we further verified these observations by analyzing the coexpression of lncRNA-IGFB4 and c-Myc in TCGA cancer samples. Our observations revealed that in most cancer types, the expression of lncRNA-IGFB4 was negatively correlated with c-Myc expression (Figure 4(c)). We specifically recapitulated this negative correlation in CESC samples with an *R* value of -0.04 (Figure 4(d)). Probably, due to the low sample size, we did not observe any significant difference (*P* value =0.44) in lncRNA-IGFB4 expression in CESC samples.

### 3.5. The Depletion of lncRNA-IGFBP4 Suppresses Cell Proliferation In Vitro

**Figure 5:**
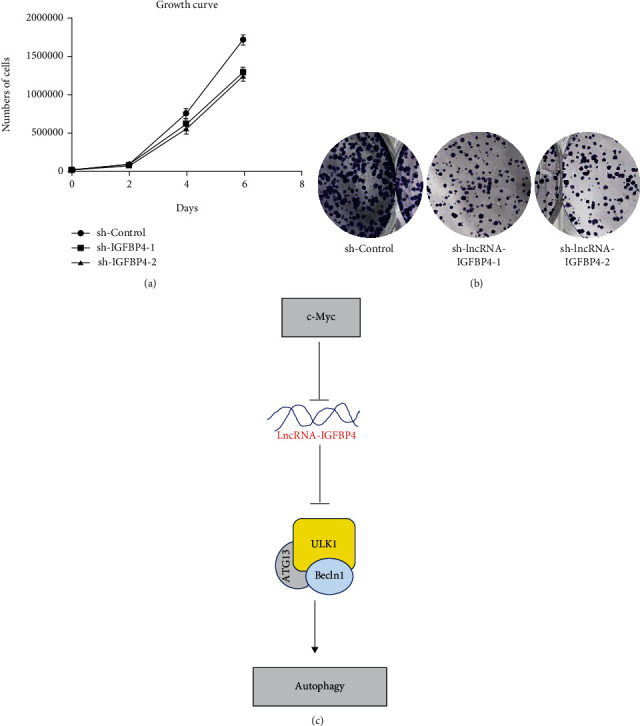
lncRNA-IGFBP4 controls the growth of HeLa Cells. (a) Cellular growth curve analysis shows reduced growth of cells in the lncRNA-IGFBP4 knockdown groups. (b). The colony formation assay showed a reduced number of colonies in the lncRNA-IGFBP4 knockdown group. (c) Sketch of a possible mechanism of autophagy regulated by c-Myc-suppressed lncRNA-IGFBP4.

We observed in the previous result section that lncRNA-IGFB4 was upregulated in most of the TCGA cancers, revealing the oncogenic role of lncRNA-IGFB4. To this end, we depleted lncRNA-IGFB4 from HeLa cells by using the shRNA-mediated lentiviral system, and the cell growth curve was assessed. We found no significant difference in cell growth for the first 4 days; however, the difference in cell proliferation was obvious after the 6th day of knockdown of lncRNA-IGFB4 (Figure 5(a)). This observation also supports the notion of involvement of autophagy-mediated growth suppression. To complement these results, we also performed a colony formation assay, which recapitulated the findings of the cell growth assay as shown in Figure 5(b). In short, the capacity of HeLa cells to form colonies after the knockdown of lncRNA-IGFB4 was significantly reduced (Figure 5(b)). Overall, these findings conclude that depletion of lncRNA-IGFB4 suppresses the cell growth of HeLa cells.

Finally, we conclude that c-Myc negatively regulates lncRNA-IGFB4 and inhibits autophagy through ULK1/Beclin1 signaling as depicted in the model diagram (Figure 5(c)).

## 4. Discussion

Autophagy is a biological process through which cells keeps balance in the homeostasis via the lysosomal degradation during cellular development and in the stress conditions [33, 34]. It is generally considered a survival mechanism through which cells remove unwanted, misfolded, or aggregated proteins; damaged organelles; and intracellular pathogens [35, 36], and autophagic cell death is considered a nonapoptotic cell death [37, 38]. Various factors have been identified as a regulator of autophagy including proteins and lncRNAs [33, 39–41], such as XIST, MALAT1, HOXA11-AS, and SNHG6, which are the well-known regulators of autophagy [42–45].

The IGFBP family proteins are known to involve in tumor growth, progression, and drug resistance in various cancers; they majorly regulate cell proliferation and metastasis [46, 47]. Mostly, these proteins have high expression in variety of cancers which has been linked with poor prognosis, advanced-stage cancer, and poor therapeutic outcomes [48–50]. Mechanistically, IGF2BPs activate the PI3K/MAPK pathway for enhancing proliferation and invasion of the glioblastoma [51]. Similarly, lncRNA-IGFBP4-1 associated with this family promotes bladder cancer through activating the JAK/STAT signaling pathway [52], which is highly crucial for the cell growth, apoptosis, and differentiation of many cancers [53, 54]. Consistent with bladder cancer finding, lncRNA-IGFBP4-1 has been considered a key regulator of metabolism which further control the proliferation and metastasis in lung cancer, prostate cancer, colon cancer, and breast cancer [21–24]. Here in the current study, lncRNA-IGFBP4 in cervical cancer showed upregulation as compared to normal tissues. Mechanistically, upon c-Myc overexpression, the lncRNA-IGFBP4 was significantly downregulated which shows the negative correlation of lncRNA-IGFBP4 with c-Myc.

As c-Myc is well known for its potential role in cancer development and cellular autophagy, it either directly regulates cellular autophagy or indirectly through interacting with other partners such as AMBRA1 regulates autophagy through dephosphorylating c-Myc [55]. Similarly, c-Myc/miR-150/EPG5 control autophagy response to promote lung cancer [56], and G9a regulates c-Myc-induced autophagy in glioblastoma cells [57]. In addition, increasing evidence showed the vital role of lncRNAs in autophagy, dual role inducing or suppressing autophagy in cancers such as liver cancer, lung cancer, and breast cancer [58–60]. A study identified 11 autophagy-related lncRNA signatures involved in the survival and prognosis of the breast cancer [19]. Among these lncRNA LINC01016 regulates ER*α*, associated with survival and prognosis of breast cancer [61], LINC00578 showed association with lung cancer and pancreatic cancer [62, 63], and LINC01016-miR-302a-3p/miR-3130-3p/NFYA/SATB1 axis was found to regulate endometrial cancer [64]. Similarly, lncRNA SOX2 regulates autophagy-mediated neuroinflammation by activating the NLRP3 inflammasome [17]. lncRNA SNHG11 activates autophagy and the Wnt/beta-catenin pathway in gastric cancer [65]. LncRNA NBR2 inhibits tumor development by regulating autophagy in HCC [66], and lncRNA CASC9 activates autophagy-mediated cell death by AKT/mTOR pathway [67]. Based on the fact that m-Myc is a significant player in the autophagy response, also lncRNAs have shown their potential in autophagy; therefore, we decided to dig out if lncRNA-IGFBP4 has any role in autophagy. Interestingly, we found that lncRNA-IGFBP4 suppresses autophagy in HeLa cells. The silencing of lncRNA-IGFB4 induces autophagy in the cells, indicated by LC3 puncta formation in GFP-LC3 in the engineered HeLa cell line. Furthermore, western blotting revealed activation of autophagy-related markers such as upregulation of LC3, Beclin1, ULK1, and downregulation of p62 in lncRNA-IGFBP4 knockdown cells. The changes in the expression levels of these markers indicate activation of autophagy in cells lacking lncRNAs-IGFBP4. Ectopic expression of the lncRNAs-IGFBP4 produced opposite results which we believe that normally, it is upregulated in the cancer cells and tissues showing low autophagy and high tumor progression. The current study has a few limitations such as the broad picture of autophagy and other proteins making complex with lncRNA-IGFB4 is not yet clear. In addition, the validating expression level of lncRNA-IGFB4 in various cancer tissues was not possible for us, and its prognostic and therapeutic roles require extensive studies and clinical validation.

## 5. Conclusion

In summary, through the TCGA database, we found upregulation of lncRNA-IGFB4 in a variety of cancers including cervical cancer. Furthermore, we verified the expression of lncRNA-IGFB4 in cervical cancer cell lines and found that lncRNA-IGFBP4 was negatively regulated by c-Myc that was analyzed by c-Myc knockdown and further luciferase reporter assay. In addition, the silencing expression of lncRNA-IGFB4 upregulates the expression of known autophagy markers. Based on these findings, we conclude that lncRNA-IGFB4 is an important factor required for the regulation of cervical cancer and c-Myc maintains cellular homeostasis through negatively regulating lncRNA-IGFB4 cervical cancer cells. Our findings provide a possibility that lncRNA-IGFB4 may potentially be used as a diagnostic and therapeutic marker in cervical cancer.

## Data Availability

Data generated by this study could be asked from corresponding author with reasonable request.

## Supplementary Materials

Supplementary Materials The list of primers (forward and reverse) used in this study was given in Supplementary Table S1.
