# Supplementary material for: c-Myc-Regulated lncRNA-IGFBP4 Suppresses Autophagy in Cervical Cancer-Originated HeLa Cells
Source: Dis Markers. 2022 Aug 29;2022:7240646. doi: 10.1155/2022/7240646 (PMC9444448; doi:10.1155/2022/7240646)
Supplement: Supplementary Materials — The list of primers (forward and reverse) used in this study was given in Supplementary Table S1. [file 7240646.f1.docx]

Table S1. Primers

| qPCR primers | Forward | GGTGGGGATGTTTGATTT |
| --- | --- | --- |
|  | Reverse | CCTGTTTTATGGGCTGAT |
| Luciferase assay | Forward | ATCTCGGCTCACTGCAACTT |
|  | Reverse | AGCCACAGTTTCCTGTCTGG |
| ChIP primers | Forward | CAGGCTTGCCTCGTACTACTG |
|  | Reverse | TGAGTCACAGCCAGCATTTT |
